# Functional and epitope specific monoclonal antibody discovery directly from immune sera using cryo-EM

**DOI:** 10.1126/sciadv.adv8257

**Published:** 2025-08-15

**Authors:** James A. Ferguson, Sai Sundar Rajan Raghavan, Garazi Peña Alzua, Disha Bhavsar, Jiachen Huang, Alesandra J. Rodriguez, Jonathan L. Torres, Maria Bottermann, Julianna Han, Florian Krammer, Facundo D. Batista, Andrew B. Ward

**Affiliations:** ^1^ Department of Integrative, Structural and Computational Biology, The Scripps Research Institute, La Jolla, CA 92037, USA.; ^2^ Department of Microbiology, Icahn School of Medicine at Mount Sinai, New York, NY 10029, USA.; ^3^ Center for Vaccine Research and Pandemic Preparedness (C-VaRPP), Icahn School of Medicine at Mount Sinai, New York, NY 10029, USA.; ^4^ The Ragon Institute of Mass General, MIT, and Harvard, Cambridge, MA 02139, USA.; ^5^ Department of Pathology, Molecular and Cell-Based Medicine, Icahn School of Medicine at Mount Sinai, New York, NY 10029, USA.; ^6^ Ignaz Semmelweis Institute, Interuniversity Institute for Infection Research, Medical University of Vienna, Vienna, Austria.; ^7^ Biology Department, Massachusetts Institute of Technology, Cambridge, MA 02139, USA.

## Abstract

Antibodies are crucial therapeutics, comprising a substantial portion of approved drugs due to their safety and clinical efficacy. Traditional antibody discovery methods are labor-intensive, limiting scalability and high-throughput analysis. Here, we improved upon our streamlined approach combining structural analysis and bioinformatics to infer heavy and light chain sequences from cryo-EM (cryo–electron microscopy) maps of serum-derived polyclonal antibodies (pAbs) bound to antigens. Using ModelAngelo, an automated structure-building tool, we accelerated pAb sequence determination and identified sequence matches in B cell repertoires via ModelAngelo-derived hidden Markov models (HMMs) associated with pAb structures. Benchmarking against results from a nonhuman primate HIV vaccine trial, our pipeline reduced analysis time from weeks to under a day with higher precision. Validation with murine immune sera from influenza vaccination revealed multiple protective antibodies. This workflow enhances antibody discovery, enabling faster, more accurate mapping of polyclonal responses with broad applications in vaccine development and therapeutic antibody discovery.

## Teaser

Protein structure tool ModelAngelo enhances speed and objectivity of polyclonal cryo-EM analysis to find protective antibodies.

## INTRODUCTION

As of 2024, antibodies comprised 206 of globally approved therapeutics, reflecting their growing efficacy in treating diverse diseases (*1*). In 2018, antibodies represented 20% of US Food and Drug Administration (FDA)–approved therapeutics (*2*). Their safety in humans (*3*) and rapid clinical translation make them an attractive approach to conditions such as cancer, autoimmunity, and infectious disease (*4*, *5*). Initially found using hybridoma technology (*6*), the invention of phage display in the 1990s (*7*), followed by improvements using yeast (*8*) or mammalian displays (*9*), has revolutionized antibody discovery throughput. Single-cell B cell receptor (BCR) sequencing with or without antigen-specific sorting offers a high throughput means of isolating monoclonal antibodies (mAbs) to a specific target and providing insights into the diversity and evolution of immune responses (*10*). However, identifying epitope-specific mAbs typically marks the culmination of a lengthy screening process, where each mAb must be individually assessed for its specificity and function (*11*, *12*). Traditional methods such as enzyme-linked immunosorbent assays (ELISAs) and functional assays provide critical insights into antibody functionality and binding specificity. However, they are highly resource-intensive due to the need for large quantities of biological and viral reagents, limiting their scalability for high-throughput screening (*13*–*16*). These bottlenecks highlight the need for alternative approaches to identify epitope-specific mAbs more efficiently, particularly through advancements in BCR-sequencing data analysis (*10*, *17*). Overcoming these challenges is essential for streamlining the mAb discovery process, as it would accelerate the identification of protective epitopes and antibody responses.

Our laboratory’s development of cryo-EMPEM (cryo–electron microscopy polyclonal epitope mapping) for characterizing polyclonal antibody (pAb) responses was a foundational step forward in being able to assess which epitopes are targeted by an individual immune response to vaccination or infection (*18*–*20*). High-resolution information contained in the pAb regions of cryo-EMPEM maps can also be used to determine antibody sequences from the structure, enabling the matching of epitope specific antibodies to sample matched BCR-sequencing datasets (*21*). While our initial study (*21*) successfully determined two epitope-specific mAbs by combining cryo-EM and BCR-sequencing data, limitations remained, particularly the time-intensive map interpretation. In addition, the isolated mAbs showed poor expression yields and targeted nonfunctional epitopes on the envelope glycoprotein of HIV-1. Recognizing the need for efficiency and the removal of human bias, we have now integrated the AI-automated model builder, ModelAngelo (MA) (*22*) and its built in HMMER search capabilities (*23*), into our structure to sequence (STS) workflow, which markedly enhances the speed and accuracy of both model building and structural analysis.

Here, we outline a method for integrating MA in conjunction with polyclonal cryo-EM maps and BCR sequencing to derive epitope-specific antibody sequences. First, we benchmarked MA’s capability to build pAb models with the two polyclonal maps that were previously used to develop the manual STS method as part of a nonhuman primate (NHP) HIV-1 Envelope glycoprotein (Env) vaccination trial (*21*). Throughout the remainder of the paper, when we refer to a pAb model, we are referring to a protein structure that is representative of the polyclonal response at a specific epitope in our polyclonal cryo-EM and not a validated mAb model with a validated sequence. The antibodies synthesized based on the MA and subsequent HMMER search recommendations exhibited higher yields with comparable binding affinities compared to those developed through our previous manual STS method. We then conducted a mouse vaccination study with the neuraminidase (NA) glycoprotein of influenza virus combined with paired BCR sequencing data to test the capability of MA to determine epitope-specific antibody sequences with a new dataset. Ultimately, we produced five NA-inhibiting mAbs that were functional in vivo and protected against viral challenge. The increased speed and accuracy of this optimized approach greatly expands the efficiency and power of antibody discovery using cryo-EMPEM.

## RESULTS

### Benchmarking MA for cryo-EMPEM STS

**Fig. 1.**
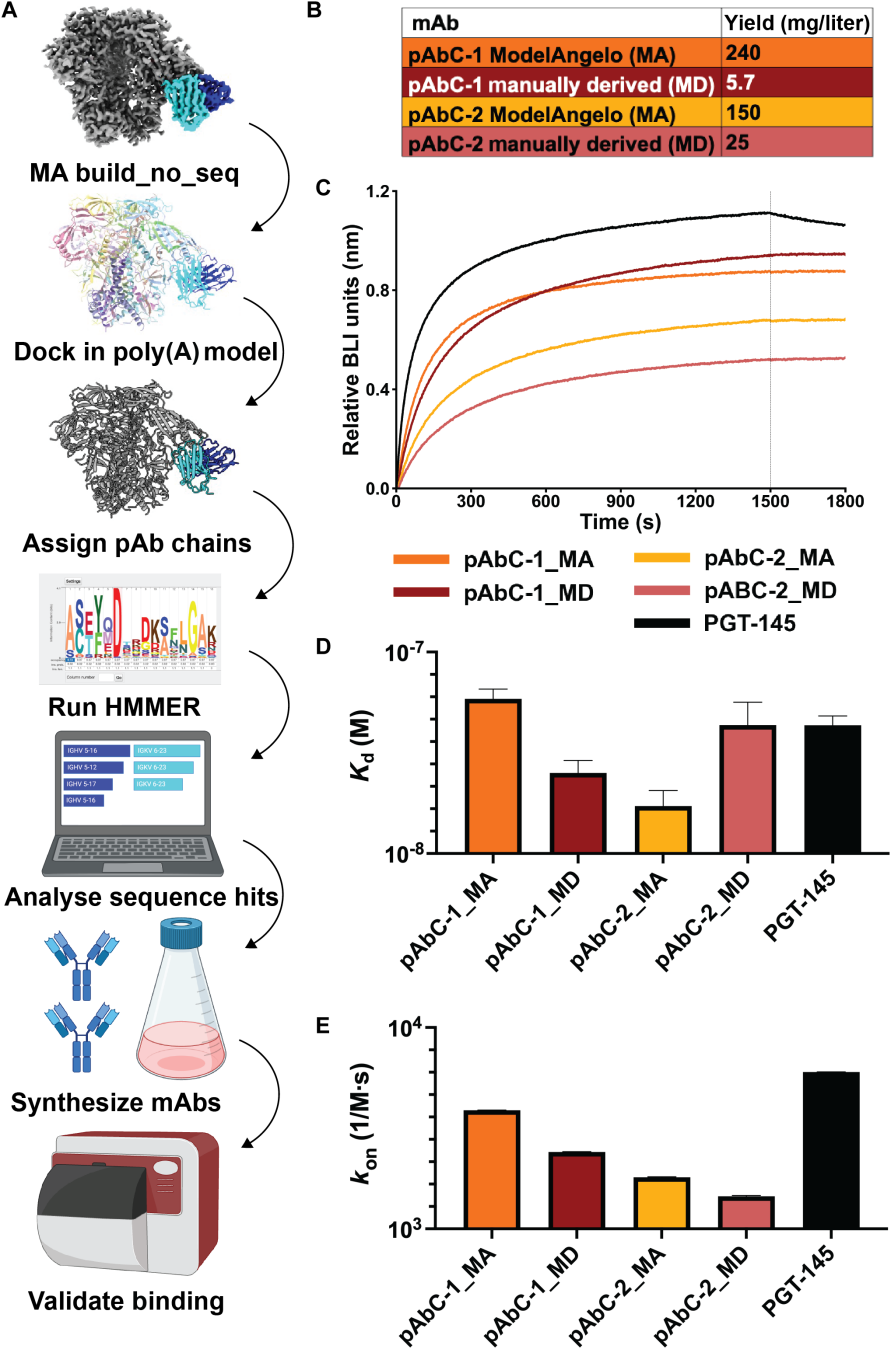
MA integrated STS workflow and benchmarking. (**A**) MA workflow. Starting with a cryo-EM map (density corresponding to the antigen shown in gray, heavy chain in blue, and light chain in cyan), MA is run in build_no_seq mode. Chains and the subsequent HMMs are assigned as heavy and light chains. A representative HMM is shown as a logo plot generated by Skylign. Heavy and light chain sequence matches are searched for in BCR sequencing repertoire using .hmm files generated by MA using the MA inbuilt HMMER. The top scoring hits are synthesized for binding validation. (**B**) Comparison of antibody yields for manually and MA-derived antibody sequences. (**C**) Representative BLI curves for highest concentration of HIV-1 Env (1000 nM). (**D**) Steady-state affinities for manually and MA-derived pAbC-1 and pAbC-2 antibodies. (**E**) On-rates for manually and MA-derived pAbC-1 and pAbC-2 antibodies. MD, manually derived. Poly(A), polyadenylate.

To benchmark MA as a pAb building tool, we ran its build_no_seq mode with the maps from our first STS dataset from NHPs vaccinated with soluble BG505 HIV-1 Env glycoprotein with the SOSIP-stabilizing mutations (*19*, *21*, *24*). This dataset included two cryo-EMPEM maps of pAbs—Rh.33104 pAbC-1 (pAbC-1) and Rh.33172 pAbC-2 (pAbC-2). pAbC-1 targets the glycan hole, and pAbC-2 targets a strain-specific epitope in the V2/V3 region, as was confirmed by the original STS paper (*21*). For both cryo-EM maps, MA generated potential pAb models and hidden Markov model (.hmm) files (Fig. 1A and fig S1). While MA built a complete heavy and light chain for pAbC-1, the pAbC-2 models were fragmented in both heavy and light chains (fig. S1). For both pAb models, the amino acid assignments did not accurately represent an antibody sequence, with an average sequence identity of 58.9% to the antibody sequences confirmed in the original STS paper (fig. S2). Because MA generates an .hmm file for each chain constructed—visualized as a logoplot in Fig. 1A generated by Skylign (*25*)—we used these instead to search for mAbs. To generate an .hmm that represents the complete heavy and light chain of the fragmented pAbC-2, we generated auxiliary code that would merge fragmented .hmm files together. This merging was guided by aligning a polyalanine Fab (fragment antigen-binding) model to the MA pAb model so that we could determine the correct order of fragments to merge. Using the heavy and light chain .hmm files that we generated from our maps, we ran MA’s HMMER search on the corresponding B cell repertoire for epitope-specific heavy and light chains. For both pAbs, we selected heavy and light chain sequences with the lowest *E*-value assigned by HMMER. Also known as the expectation value, HMMER assigns an *E*-value to each sequence in a user’s search database and is a measure of statistical significance for that sequence. The *E*-value represents the probability of finding that sequence by chance within that search database (*26*). These putative heavy and light chain sequences had an average of 92.3% identity to the original manually derived STS mAbs (fig. S3). We then expressed these Abs to validate binding.

Before considering affinity, we observed that MA-derived antibodies showed higher yields than the manually derived sequences (Fig. 1B). For the human manually derived mAbs, pAbC-1_HU and pAbC-2_HU, we got yields of 5.7 and 25 mg/liter, respectively. Whereas for the MA-derived mAbs, pAbC-1_MA and pAbC-2_MA, the yields were 240 and 150 mg/liter, respectively. We measured affinities by biolayer interferometry (BLI), and because no measurable off rate could be determined (Fig. 1C and fig. S4), for all mAbs, we compared the steady-state affinities and on rates (Fig. 1, D and E, respectively). For pAbC-1, the manually derived mAb had a marginally higher affinity with an apparent steady-state *K*_d_ (dissociation constant) of 25 nM compared to the MA-derived mAb with 59 nM. However, for pAbC-2, the human-derived mAb had a *K*_d_ of 43 nM, which was a lower affinity than the MA-derived pAbC-2 with *K*_d_ of 17 nM. When looking at the on-rates, the situation is reversed. For pAbC-1, the MA-derived mAb had an on rate of 3.9 × 10^−3^ M^−1^ s^−1^, which was higher than the human-derived mAb with an on rate of 2.4 × 10^−3^ M^−1^ s^−1^. For pAbC-2, the manually derived mAb bound faster with on rate of 1.8 × 10^−3^ M^−1^ s^−1^ compared to the MA-derived mAb at 1.4 × 10^−3^ M^−1^ s^−1^. These results highlight that MA can rapidly identify candidate antibodies with higher yields and comparable binding profiles in under 2 hours compared to days or weeks of manual analysis of polyclonal cryo-EM maps. Specifically, MA runs typically complete in under 30 min on an RTX A5000 GPU, with the remaining time used for interpretation of the MA output and HMMER-based sequence analysis. In contrast, manual approaches require labor-intensive residue-level interpretation and the construction of putative full-length antibody sequences, which is both time-consuming and less scalable.

### Testing MA on an influenza virus NA vaccination study in mice

**Fig. 2.**
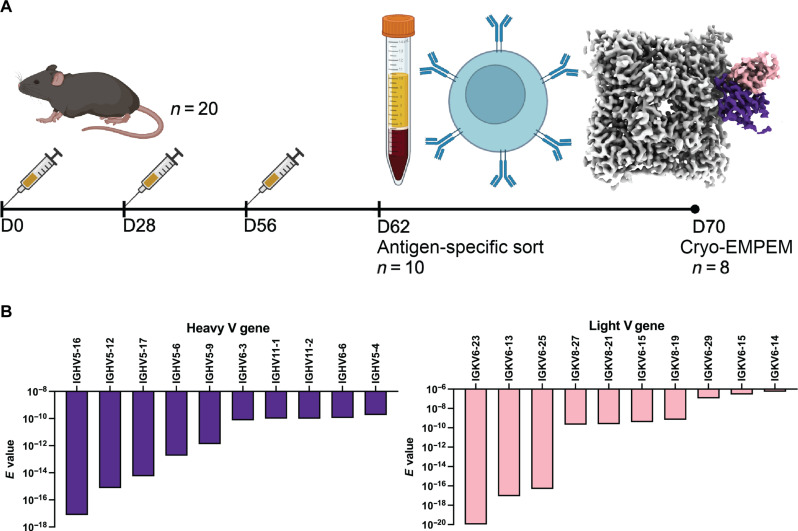
Predicted active site pAb gene usages after NA vaccination. (**A**) Vaccination scheme for cryo-EMPEM and B cell sorting. Density corresponding to the heavy and light chains of the active site pAb is shown in purple and pink, respectively. The NA density is shown in light grey. (**B**) Top gene usages for the heavy and light chains of the active site region pAb.

With the method successfully benchmarked, we tested the workflow to find functional influenza antibodies from mice vaccinated with NA from A/Indiana/10/2011 (Ind11) H3N2 (Fig. 2A). Cryo-EMPEM analysis on sera pooled from 10 mice revealed robust antibody responses against multiple NA epitopes (fig. S5). Because we were interested in functional antibodies, we specifically generated a 3.3-Å polyclonal cryo-EM map with a pAb binding the active site of NA with our processing workflow shown in fig. S5 and data collection statistics in table S1. We ran MA build_no_seq on this map to build an initial model (fig. S6) and .hmm files. As with the pAbC-2 benchmarking map, the MA model of the active site pAb was fragmented (fig. S6). Likely due to the polyclonality and multi-animal pooled sera of the active site binding pAb responses, the cryo-EM reconstruction of the pAb HCDR3 regions was ambiguous, and the assignment of Cα positions by MA was incorrect so we manually determined pAb loop lengths and made sure that our merged .hmm reflected this. Hence, we inserted three additional positions with random amino acid probabilities into the CDRH3 and CDRH2 in the FW1 and FW3 regions in the heavy chain .hmm as demonstrated in fig. S6.

For this batch of 10 mice, we were unable to obtain BCR sequences. In their absence, we used the V gene germline database for C57BL/6 mice from the International Immunogenetics system (IMGT) (*27*) as an input for HMMER search to determine the gene usage of the pAb (Fig 2B). The HMMER E-value was used to rank the gene usage. Based on the E-values, the top five most likely IGHV gene usages were IGHV5-16, IGHV5-12, IGHV5-17, IGHV5-6, and IGHV5-9. For the light chain, the top five most likely IGLV or IGKV gene usages were IGKV6-23, IGKV6-13, IGKV6-25, IGKV8-27, and IGKV8-21.

**Fig. 3.**
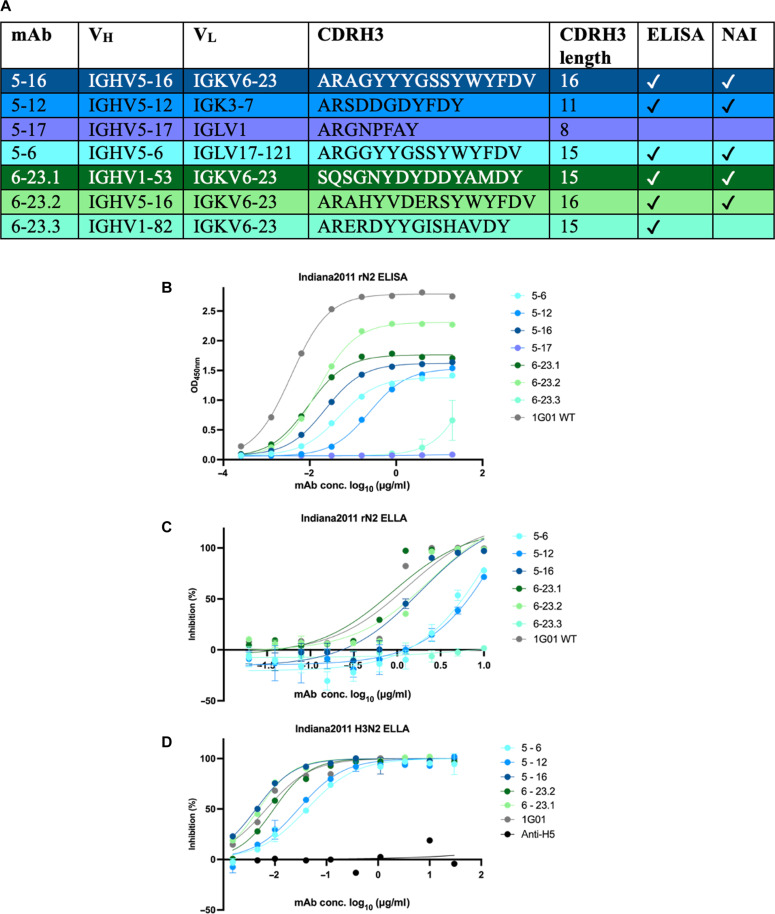
Predicted active site mAbs bind and inhibit NA activity. (**A**) Table of selected mAbs from HMMER search. NAI, neuraminidase inhibition. (**B**) ELISA of selected mAbs against Ind11 NA. (**C**) ELLA of selected mAbs against recombinant Ind11 NA. (**D**) ELLA of selected mAbs against live Ind11 influenza virus. The well-defined anti-NA antibody was used as a positive control (1G01). WT, wild type; OD_450nm_, optical density at 450 nm.

Another eight mice underwent the same vaccination scheme, and splenocytes were harvested to generate a database of roughly 5000 NA-specific paired antibody sequences for our HMMER search to find active site epitope-specific mAbs. After completing the heavy chain HMMER search many of the lowest E-value sequences used the IGHV5-16 V gene predicted in Fig 2B. However, we were also curious whether sequences using the other likely V genes—such as IGHV5-12, IGHV5-17, and IGHV5-6—might also bind to the active site epitope. Hence, we picked four mAbs with the lowest E-value each using a different one of the top four V genes displayed in (Fig. 2B). After completing the light-chain HMMER search, many of the lowest E-value sequences used the IGKV6-23 V gene predicted in Fig. 2B. However, we noticed that there was a variety of heavy V genes paired with these light chains, including many that were not suggested to be used in Fig. 2B; hence, we chose these three sequences with the lowest E-values, all using the most likely light-chain gene IGKV6-23 (Fig. 2B); however, each of these mAbs used a different heavy-chain V gene (Fig. 3A). These seven mAbs that were chosen for further validation are listed in Fig. 3A. Six of the seven selected mAbs bound to Ind11 NA by ELISA (Fig. 3B), and five inhibited recombinant Ind11 NA using an enzyme-linked lectin assay (ELLA) (Fig. 3C). Before conducting protection studies, we confirmed that these mAbs inhibited NA in the context of live Ind11 influenza virus (Fig. 3D).

### Structural epitope analysis of NA-inhibiting mAbs

**Fig. 4.**
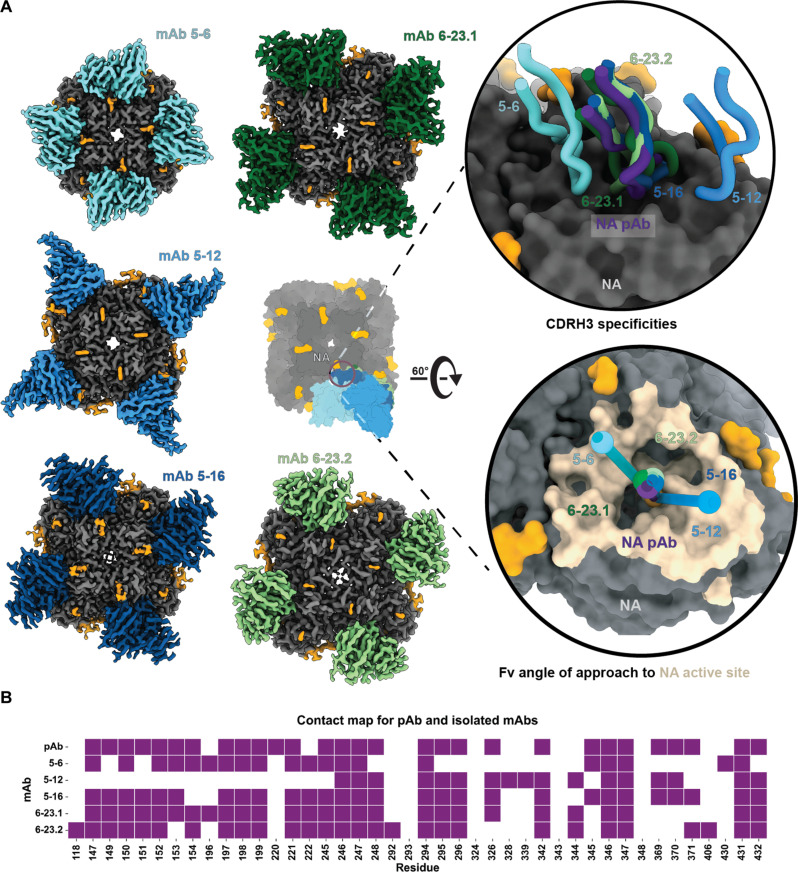
Structural comparison of epitope:paratope interface of NA pAb and MA-isolated mAbs. (**A**) CryoEM maps of the five isolated mAbs including a peanut model overlay of the mAbs. The top inset of the active site compares the backbones of the CDRH3 regions. The bottom inset compares the angle of approach of each mAb. The angle is measured between the mAb centroid, the active site centroid, and the pAb centroid. (**B**) Residues that contributed more than 5 Å^2^ of BSA due to mAb or pAb binding. The list of residues listed was determined using the GetContacts interface analyzer tool.

To map the epitopes targeted by the five NA-inhibiting mAbs, we solved EM structures of these mAbs in complex with the Ind11 NA, with resolutions ranging from 2.8 to 3.4 Å (Fig. 4A and fig. S7), and validation metrics and CDRH3 densities shown in table S1 and fig. S7, respectively. To compare the isolated mAbs with the polyclonal cryo-EM map, we built a representative pAb model. Because the polyclonal cryo-EM map represents a heterogenous antibody response with variable amino acids being represented and a high diversity of putative sequences, particularly in CDR regions, building a representative model was challenging. We decided to build a pAb model using the germline IGHV5-16 and IGHJ1 sequences with a CDRH3 length of 16 and light chain using the germline IGKV6-23 and IGKJ3. We used Ablang2 (*28*) to predict the eight missing CDRH3 residues that would have been inserted during VDJ recombination, and then built the pAb model using Abodybuilder2 (*29*), followed by manual fitting in COOT (*30*) with the NA model.

Using the protein interface analyzer tool, getcontacts (*31*), we determined all NA contact residues used collectively by the pAb and the five mAbs, shown in red on the NA surface in Fig. 4A and listed in the contact plot in Fig. 4B. To assess similarity, we compared the buried surface area (BSA) of each of the NA contact residues for each mAb to the pAb using Biopython (*32*), normalizing against unbound NA. Residues contributing more than 5-Å BSA are shown in Fig. 4B with a normalized BSA heatmap in fig. S8A. These results indicated that that 5-16 is most structurally similar to the pAb, differing by only three contact residues, while 5-12 differed by 17 of 42 collective residues. Pearson’s correlation coefficients for normalized BSA (fig. S8B) suggest that 5-16 (0.93) and 6-23.1 (0.89) most closely resemble the pAb. Root mean square deviation (RMSD) Cα after model alignment to the pAb bound protomer showed 5-16, 5-6, and 6-23.2 has the lowest Cα RMSDs (1.7 Å), while 6-23.1 and 5-12 had an RMSD of 2.7 Å. Last, we compared the angle of approach of each mAb compared to the pAb (Fig. 4A). The angle of approach calculations revealed that all mAbs had less than 3° difference from the pAb except, for 5-12 and 5-6, which had differed by 28°. Overall, our structures suggest that 5-16 and 6-23.2 are the most structurally representative of the active site pAb that we identified in terms of contacts, BSA contribution, and RMSD Cα, and these mAbs also exhibit the greatest inhibition potency to live virus.

### Protection study in mice

**Fig. 5.**
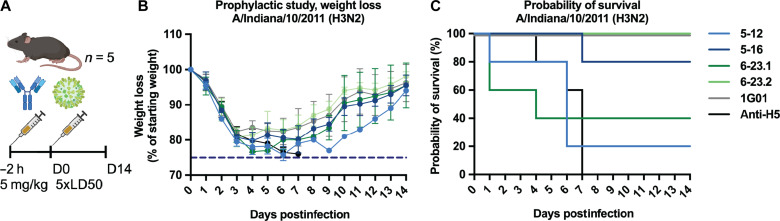
Prophylactic protection study using MA-isolated NA-inhibiting mAbs. (**A**) Six-week-old female DBA/2J mice (*n* = 5 mice per group) were injected intraperitoneally with generated mAbs (5 mg/kg; 5-12, 5-16, 6-23.1, and 6-23.2), a well-defined anti-NA antibody was used as a positive control (1G01), and an irrelevant antibody (anti-H5) was used as a negative control. The antibodies were administered 2 hours before the mice were infected with 5 times LD_50_ (lethal dose 50%) of the A/Indiana/10/2011 (H3N2) virus. The percentage of initial body weight (**B**) and survival (**C**) was monitored and plotted over a 14-day period postinfection for each antibody group. The percentage of body weight is relative to the starting body weight, and the data are represented as group means with SD. (C) 1G01 data are offset by 1% for clarity.

To evaluate whether the generated mAbs using the developed method could inhibit viral replication in vivo, we tested the different antibodies in a prophylactic setting. Six-week-old DBA/2J mice were administered with mAbs (5 mg/ml) 2 hours before intranasal infection with Ind11 H3N2 virus at a dose of five times the lethal dose 50% (LD_50_). Mice were then monitored for weight loss and survival for 14 days to assess protection (Fig. 5). The antibodies with greatest NA inhibitory activity in vitro (5-16, 6-23.1, and 6-23.2) also showed better protection from infection in mice, with those treated with 6-23.2 achieving complete survival similar to mice administered with positive control mAb 1G01, while mice receiving 5-16 and 6-23.1 had survival rates of 80 and 66.7%, respectively. This degree of protection was not seen in mice administered with 5-12 or with the irrelevant anti-H5 mAb, indicating that our MA-powered STS pipeline can predict and facilitate the generation of mAbs with effective protective capacity.

## DISCUSSION

As was demonstrated in our original STS manuscript and subsequent studies (*18*, *20*, *21*, *33*), the single-particle nature of cryo-EM enables the effective examination of the heterogeneous immune response to vaccination and infection. Extracting sequence information from pAb cryo-EM maps is a powerful immunological tool for identifying epitope-specific antibodies within sequence repertoires (*21*). The integration of MA into this workflow has enhanced this pipeline as demonstrated well with our benchmarking dataset and with more recent analyses (*34*, *35*).

First, it has sped up the analysis of cryo-EMPEM maps to under 2 hours meaning that data analysis is no longer a bottleneck. Second, unlike our previous manual STS analysis, MA generates .hmm files for faster, unbiased, probabilistic sequence searches, although having to manually merge .hmm files together and determine potential missing residues introduces some human bias. Despite this introduced bias, we and the Snijder group (*33*) observe that working with single small .hmm files or sequences fragments of a pAb, the best result achievable is typically a V gene assignment. Last, using HMMER for exhaustive, bias-free searches has transformed the STS pipeline into a high-throughput scalable tool for epitope-specific antibody discovery.

The reduction in human bias of amino acid assignment of putative mAb sequences likely contributed to the improved yields for the HIV-1 benchmarking pAbs, notably pAbC-1. Our original manual method involved manually assigning each Cα and side-chain identity at each position, whereas MA systemically assigns Cα and an amino acid probability at each position ultimately opening the search space for the most representative mAb sequence from the polyclonal map. The unpaired BCR sequencing repertoires for the NHPs makes selecting a pair of functional sequences challenging. For pAbC-1, the MA-derived heavy chain, vs. the human derived, had only two amino acid substitutions occurring in the framework regions. However, the light chain had 18 differences, half of which occurred in framework region two. Given the location of these substitutions, it suggests that a large part of the yield improvements stem from improved mAb stability, particularly in the heavy-light chain interface.

Regardless of whether STS is done manually or with MA a major limitation remains the quality of the cryo-EM map. Ambiguities arise from three main sources: sample specific issues leading to poor resolution of orientation bias, inherent flexibility of the pAb constant domains causing locally lower resolutions, and last, the polyclonal nature of pAb maps, meaning that maps represent a composition of amino acids. Each of these pathologies can result in MA generating fragmented chains that we saw in pAbC-2 and our NA immunization dataset. The need to merge .hmm files and identify missing residues between chains introduces human bias and extra analysis time. In the future, it may be possible to use other probabilistic tools to determine these ambiguous regions. For example, inverse folding tools such as Antifold (*36*) or ProteinMPNN (*37*), with retrained weights (*38*), or fine-tuned antibody language models (*28*) may be used in combination with initial protein structure models to predict probabilistic residues in regions deemed to be ambiguous. Even fine-tuning of the MA model itself on only antigen:antibody structures may improve the accuracy of resultant .hmm files. Recently, it has been possible to design functional antibodies de novo using diffusion models that could aid our discovery pipeline (*39*). Ultimately, protein design tools generate putative binding sequences that could be down selected for sequence matches as we have done here with MA or even used as search databases using the Snijder group’s mass spectrometry based to find de novo epitope-specific antibodies (*33*).

In our NA immunization dataset, we introduced three random amino acids in the CDRH3 and CDRH2 in both FW1 and FW3 in our heavy .hmm to address polyclonality. Despite these ambiguities, we found five functional mAbs all targeting the same epitope, with two that were able to inhibit as potently as the best in class mAb 1G01 (Fig. 3D) (*40*). As postulated in the original STS paper the complete sequence of the pAb may not be needed to find functional antibodies. Notably, two of the five neutralizing mAbs were discovered using only the light chain sequence alone—thanks to the paired BCR sequencing dataset. mAb 6.23-1, found via the light chain, used the unexpected IGHV1-53, contrary to the predicted V gene usages shown in Fig. 2. The most potent inhibitor, mAb 5-16, closely resembled the pAb in the polyclonal map, a trend that continued for the remainder of the isolated mAbs except for 6-23.1 and 6-23.2, where the former is more potent but slightly less similar to the pAb. The trend further follows with the protection data with 6-23.2 protecting 100%, 5-16 protecting 80%, and 6-23.1 protecting 66%. The 5-12, which structurally diverges from the pAb the most, only protected 25% of the mice, and 5-6 did not protect at all; however, for this mAb, we did not generate a statistically significant protection result. While STS is not a quantitative method, these findings demonstrate how cryo-EMPEM can capture the most abundant and tightest binding pAbs with protective properties.

Cryo-EM is a valuable tool for understanding the structure-function relationship of protective mAbs and is now a pivotal tool in the understanding of serum immune responses through characterizing pAb sequences from cryo-EM maps. Its single-particle nature could allow for synergistic integration with quantitative techniques such as proteomics-based method Ig-seq (*41*, *42*). It may also be useful in finding public clonotypes as demonstrated in this study, where not only were the mice that were sequenced not the same mice that the pAb map came from, the pAb map also represents the composite response of 10 mice. Further, the cryo-EMPEM maps enable us to focus in on serum mAbs to specific functional epitopes, such as the NA active site, which contrasts with hybridoma or antigen-specific B cell sorting strategies that generate antibodies in an epitope agnostic manner. Hence, the efficiency of on-target antibody discovery is thereby increased.

Machine learning tools will without doubt continue to improve the speed and quality of immune repertoire analysis, which is a vital part of structure-based antibody and vaccine design (*43*). Here, we show how the machine learning tool MA has revolutionized our STS workflow, rapidly deconvoluting pAb responses with speed and precision. This integration of ML into cryoEM-based immune profiling not only accelerates vaccine development but also enhances our ability to respond swiftly to emerging pathogens. By streamlining the discovery of antigen-reactive and protective antibodies, this pipeline paves the way for faster, epitope-specific antibody identification in the face of future global health threats.

## MATERIALS AND METHODS

### Expression and purification of BG505 SOSIP and PGT145

Antigen expression and purification were performed as described previously (*17*). Briefly, BG505 SOSIP.664 was expressed in HEK293F cells (Thermo Fisher Scientific). The proteins were purified from cell supernatants using PGT145 immunoaffinity chromatography (*44*). The 3 M MgCl_2_ buffer was used for protein elution from the immunoaffinity matrix. BG505 SOSIP.664 samples were concentrated, buffer-exchanged to tris-buffered saline (TBS) (Alfa Aesar) and subjected to size exclusion chromatography (SEC). HiLoad 16/600 Superdex 200 pg (GE Healthcare) running TBS buffer was used for SEC purification. Fractions corresponding to the BG505 SOSIP.664 trimer were pooled, concentrated to 1 mg/ml, and frozen for storage.

PGT145 IgG was expressed in HEK293F cells (Thermo Fisher Scientific) by cotransfecting plasmids containing IgG heavy chain, light chain, and TPST2 (tyrosylprotein sulfotransferase). Recombinant IgG was purified from cell supernatants using a prepacked MabSelect protein A affinity column (Cytiva), eluted with 0.2 M glycine (pH 2.0), buffer-exchanged to TBS buffer, and frozen.

### Expression and purification of N2 A/Indiana/10/2011 NA antigen

A/Indiana/10/2011 N2 was expressed by BioMetas using a modified version of a protocol previously described here (*45*). Briefly, NA was expressed in Expi293 cells using a Twin-Strep-Thrombin-hVASP-NA-ectodomain construct. Proteins were purified from cell supernatants using TwinStep column, concentrated, and purified further using a SEC column. Fractions corresponding to NA were pooled, concentrated, and frozen for storage.

### Immunization experiments

C57BL/6J mice (8 to 10 weeks old; the Jackson Laboratory, RRID:IMSR_JAX:000664) were immunized intraperitoneally with 10 μg of N2 (A/Indiana/10/2011, Biortus Biosciences Co. Ltd., BioMetas) formulated in Sigma Adjuvant System (Sigma-Aldrich) and boosted on days 28 and 56. Eight mice were terminated on day 70 for spleen collection and 10 mice on day 62 for submandibular blood collection into BD MicroTainer Blood Collection Tubes (Beckton-Dickinson). Serum was collected after centrifugation (5 min, room temperature, 20,000*g*). All immunization experiments were performed under Harvard University and Massachusetts General Hospital (MGH) International Animal Care and Use Committee (IACUC)–approved protocols (2016 N000286) in Association for Assessment and Accreditation of Laboratory Animal Care-accredited MGH facilities.

### BCR sequencing: Fluorescence-activated cell sorting

Mono-biotinylated N2 (A/Indiana/10/2011, BioMetas) was incubated with streptavidin (Streptavidin-AF647 and Streptavidin-PE, BioLegend) in a 4:1 ratio at least 30 min before staining. Splenocytes were enriched for activated B cells. Enriched splenocytes were stained in 50 nM probe mix (assembled as described above) for 30 min, followed by staining with fluorescent antibody mix (CD38-BV510, CD138-BV605, IgD-BV786, B220-FITC, CD95-PE-Cy7, NK1.1-APCeF780, Gr-1-APCeF780, F4/80-APCeF780, CD4-APCeF780, an d CD8-APCeF780), TotalSeq-C antibody mix (TotalSeq-C0914, CD273; TotalSeq-C0557, CD38; TotalSeq-C0917, CD95; TotalSeq-C0810, CD138; TotalSeq-C0571, IgD), and TotalSeq C hashtags (BioLegend) for 30 min. Cells were resuspended in phosphate-buffered saline (PBS) + 2% fetal bovine serum + 4′,6-diamidino-2-phenylindole (200 ng/ml) (Thermo Fisher Scientific). Cell sorting was performed on a BD FACSAria Fusion (BD Biosciences).

### BCR sequencing: Cell encapsulation and sequencing

Cell encapsulation was performed using a Chromium Controller (10x Genomics) and the Chromium Next GEM Single Cell 5’ Kit v2. Libraries were built according to the manufacturer’s instructions. Libraries were sequenced using a NextSeq2000 Sequencing System (Illumina) as previously described (*46*).

### BCR sequencing: Single-cell RNA sequencing analysis

Sample preprocessing was performed using the Cell Ranger analysis pipeline (v7.2.0, 10x Genomics) aligning to the mm10-2020-A reference transcriptome. Sample demultiplexing, quality control, normalization, and clustering were performed with the R package Seurat v4.3.0 (*47*).

### Isolation of mouse sera IgG and Fab preparation for cryo-EMPEM

EMPEM protocols were performed as described by the EMPEM with deviations described below (*48*). Serum was heat-inactivated in a 56°C water bath for 60 min and filtered with a 0.2-μm filter, and Triton X-100 was added to a final concentration of 1% v/v. Total IgG was purified using a Cytiva ALIAS autosampler and Cytiva AKTA pure system using a prepacked HiTrap MabSelect PrismA column (Cytiva). IgG was eluted with by incubation with 0.1 M glycine (pH 3.0) directly into wells containing 1 M tris-HCl (pH 8.0) buffer. Samples were buffer-exchanged into PBS using centrifugation with 30 kDa cutoff Amicon concentrators. Total IgG was digested with papain in freshly prepared digestion buffer [20 mM sodium phosphate, 10 mM EDTA, and 20 mM cysteine (pH 7.4)] at 37°C for 5 hours and was quenched with excess iodoacetamide. Samples were buffer exchanged into PBS using 10 kDa cutoff Amicon concentrators, and Fc fragments were removed with Capture select resin. To purify any further serum impurities and papain, the polyclonal fab mixture was run through size exclusion chromatography with a Superdex 200 increase 10/300 column (GE Healthcare) in TBS. Fab-rich fractions were pooled and concentrated for EMPEM complex preparation.

### Preparation of Fab NA polyclonal complexes

A/Indiana/10/2011 N2 (60 μg) was incubated with polyclonal fab (2 mg) at room temperature overnight. The complex was SEC-purified using a HiLoad 16/600 Superdex 200 pg (GE Healthcare) column, with TBS as a running buffer. SEC fractions corresponding to the complex were pooled and concentrated to 0.5 mg/ml using an Amicon filter unit with 10 kDa cutoff (EMD Millipore)

### Preparation of grids for cryo-EMPEM and imaging

UltrAuFoil 1.2/1.3 grids (Au, 300 mesh; Quantifoil Micro Tools GmbH) were used for sample vitrification. The grids were treated with Ar/O_2_ plasma (Solarus 950 plasma cleaner, Gatan) for 25 s at 15 mA immediately before sample application. A total of 0.5 μl of 0.7% (w/v) octyl-beta-glucoside (OBG) was added to 3.0 μl of the complex, and 3 μl at 0.5 mg/ml was immediately loaded onto the grid. Grids were prepared using Vitrobot mark IV (Thermo Fisher Scientific). The temperature inside the chamber was maintained at 4°C, while humidity was at 100%. Blotting force was set to 1 and wait time to 4.5 s. Following the blotting step, the grids were plunge frozen into liquid ethane and cooled by liquid nitrogen. Grids were loaded into a Thermo Fisher Glacios microscope operating at 200 kV. Movies were collected at ×190,000 nominal magnification using a Falcon 4i camera using automated image collection software EPU 3.5.1 (Thermo Fisher Scientific). Movies were aligned, dose-weighted, and CTF-corrected in the CryoSPARC Live software platform.

### Cryo-EMPEM data processing

Data processing was carried out in CryoSPARC v4.5.1. Particles were picked using Template picker with a 200-Å particle diameter using templates generated from class averages from CryoSPARC Live two-dimensional (2D) classification jobs (*49*). Clean particles stacks were selected from further reference-free 2D classification jobs. These clean particles stacks were downsampled from their initial box size of 512 to 64 pixel using a downsample particles job and underwent ab initio reconstruction requesting 10 classes with a maximum alignment resolution of 20 Å. Four immune complex volumes and one junk volume were selected for a subsequent heterogenous refinement with initial alignment resolution of 25 Å and maximum alignment resolution of 20 Å. Particles from the classes with active side–binding antibodies were re-extracted at a box size of 512 pixel and underwent nonuniform refinement (*50*), followed by local refinement using a NA-fv (fragment variable) mask generated from a Volume Tools job using an input volume generated in Chimera (*51*) with molmap and mask parameters: 0.05 map threshold, 3-pixel dilation radius, and 6 pixel soft padding width. This final map was used for the MA analysis.

### Cryo-EMPEM model building with MA

For HIV-1 benchmarking, previously published maps were used and obtained from the Electron Microscopy Databank (EMDB) as follows: EMD-23227 for pAbC-1 and EMD- 23232 for pAbC-2. For the NA cryo-EMPEM reported here, the map has been deposited in EMDB with ID no. EMD-48118. For all experiments reported here, MA v1.0.1 was used. All maps were run using the “model_angelo build_no_seq” command with default parameters. In many cases, the chains and .hmm profiles that were output by MA were fragmented. In these cases, a polyadenylate fv model was aligned with the output.cif file in Pymol or ChimeraX (*52*, *53*) to determine which .hmm profiles should be merged together to form a complete heavy or light chain .hmm profile. To merge .hmm files generated by MA, we generated auxiliary code that generates an amino acid probability array from the individual .hmm files from the fragments that can be merged into one complete array. Then, using the framework of the MA code, aaprobstohmm.py (*22*), that is responsible for generating .hmm files from the amino acid logits generated in an MA build_no_seq run, a new .hmm of the merged fragments can be generated. When it was deemed that an amino acid was missing between fragments, we inserted a random amino acid into the .hmm file (5% probably of each of the 20 amino acids). A list of fragments that were pieced together can be found in this paper’s github repository, and using the NA pAb model as an example, this workflow is demonstrated in fig. S7. The output model’s .hmm profiles were used to search the unpaired BCR sequence database using HMMER (*26*).

### MAb selection using MA’s HMMER functionality

For HIV-1 benchmarking data, where BCR sequencing data were unpaired, the heavy and light sequences with the lowest E-value were selected for mAb synthesis. For the NA vaccination study, the heavy chains sequences with lowest E-values for the top four predicted heavy V gene usages shown in Fig. 2B were selected and for the light chain, and three IGLV6-23 sequences with the lowest E-values and unique IGHV gene pairs were selected for mAb synthesis. Upon inspection of the top hits for IGHV5-6 and IGHV5-17 built into the cryo-EMPEM map, we decided that the second lowest E-value sequence for these V genes fit better and thus moved forward with these mAbs for validation.

### MAb expression

All mAbs derived from MA analysis and the STS mAbs previously described from the original STS study were expressed using GenScript’s TurboCHO v2.0 service using 10-ml expressions. Macaque fv sequences were synthesized into a human IgG1 backbone, while mice fv sequences were synthesized into a mouse IgG1 backbone.

### Biolayer interferometry

IgG-binding kinetics were measured by BLI on an Octet 96 Red instrument (ForteBio) using AHCII biosensors (Sartorius). IgGs were diluted to 5 μg ml^−1^ in kinetics buffer [20 mM Hepes (pH 7.5), 0.02% Tween 20, and 0.1% BSA]. Each analyte was serially diluted to the desired concentration in kinetics buffer with the following final concentrations: 1000, 500, 250, 125, 62.3, 32.3, and 15.6 mM. IgGs were loaded onto the biosensor for 60 s. After loading, biosensors were placed in kinetics buffer for 60 s for a baseline reading. Biosensors were then placed in analyte for 1500 s in the association phase, followed by 300 s in the dissociation phase in kinetics buffer. The background signal from an unloaded sensor that underwent the same cycle with an analyte well of 1000 mM was subtracted from the other seven signals. Kinetic analyses were performed twice with independently prepared analyte dilution series. Curve fitting was performed using a 2:1 binding model using the Octet data analysis studio (v13.0.2.46). Final plots were prepared using Matplotlib.

### Virus production

A/Indiana/10/2011 (Ind11) H3N2 virus was obtained from International Reagent Resource (catalog no. FR-947) and injected into 10-day-old embryonated specific pathogen–free chicken eggs and incubated for 48 hours at 37°C. At the end of the incubation period, the allantoic fluid from the eggs was harvested and clarified by centrifugation at 300*g*. The clarified allantoic fluid was then aliquoted and stored at −80°C.

### ELISAs with NA

High binding plates (96 well; SpectraPlate-96HB) were coated with recombinant NAs diluted in PBS at 2 μg/ml (100 μl per well) overnight at 4°C. Plates were washed three times with PBS with 0.05% (v/v) Tween 20 (PBST) in the BioStack Microplate Stacker system (BioTek) (same washing conditions were used for all the following steps) and then blocked (200 μl per well) with nonfat milk (5% w/v in PBST) for 1 hour (all incubations were performed at room temperature). Primary antibodies were serial diluted (starting at 20 μg/ml and followed by 1:5 dilutions) in PBST and aliquoted in plates (100 μl per well) after the plates were washed. After 1 hour, the plates were washed again and incubated with goat anti-mouse (Bio-Rad, #STAR120P) or anti-human (Invitrogen, #SA5-10283) IgG Fc–horseradish peroxidase (HRP) secondary antibodies (1:20,000 diluted in PBST, 100 μl per well) for 1 hour. Following PBST washing, the 3,3′,5,5′-tetramethylbenzidine (TMB) (Sigma-Aldrich, #CLS2592) two-component Microwell Peroxidase Substrate Kit (Seracare, #5120-0047) was used for color development according to manufacturer’s protocol. Synergy H1 plate reader (BioTek) was used for acquisition of colorimetric data by recording the absorbance at 450-nm wavelength. Data were analyzed with GraphPad Prism (version 10), and midpoint titers (median effective concentration) were determined. All experiments were performed in duplicates.

### NA ELLA with recombinant NA and Ind11 H3N2 virus

High binding plates (96 well; SpectraPlate-96HB) were coated with fetuin (25 μg/ml; Sigma-Aldrich, #3385, 100 μl per well) diluted in coating buffer (KPL, #50-84-01). To determine the optimal recombinant NA or Ind11 H3N2 virus concentrations used for ELLA, 50 μl of recombinant NA, serially diluted in sample diluent (PBS with CaCl_2_ and MgCl_2_ + 1% BSA + 0.5% Tween 20), starting from 20 μg/ml followed by 1:3 dilutions, or 150 μl of 1:2 serially diluted A/Indiana/10/2011 (Ind11) H3N2 virus was transferred to fetuin-coated plates after washing three times with PBST. The plates were then sealed and incubated at 37°C for 16 to 18 hours with the recombinant NA or were incubated at 33°C for 6 hours with the A/Indiana/10/2011 (H3N2) virus. Next, plates were washed five times with PBST before adding 100 μl per well of peanut agglutinin (1 μg/ml)–HRP (1 mg, diluted in sample diluent without Tween 20; Sigma-Aldrich, #7759) in each well. The plates were incubated at room temperature for 2 hours and then washed three times with PBST. The color was developed with the same TMB substrate kit used in ELISA above. Data were analyzed with GraphPad Prism (v10), and a standard curve was determined. The NA concentration within the linear region of the standard curve that conferred the optical density at 450 closest to the max signal was selected for ELLA (the selected signal should also be at least 10-fold higher than background signal). To determine the NA activity inhibition of the antibodies, 25 μl of serial diluted antibodies (1:2 dilution starting from 20 or 30 μg/ml) were mixed with 25 μl of NA (final NA concentration should match that was determined above). The 50-μl mixture was then transferred to each well in washed fetuin-coated plates. The rest of the ELLA assay was finished by following the same steps described above. The NA activity inhibition was quantified as: 100% − (signal of sample well/signal of NA-only well) × 100%, with the NA-only well has no inhibition (0%). Data were analyzed with GraphPad Prism (version 10), and midpoint inhibitory titers (IC_50_) were determined. All experiments were performed in duplicates.

### Mice protection experiment

Six-to-eight-week-old female DBA/2J mice from the Jackson Laboratory were used for all animal experiments. All procedures were conducted in accordance with protocols approved by the IACUC at the Icahn School of Medicine at Mount Sinai.

For the experiment, six groups of three to five mice each were administered intraperitoneally with 5 mg/mL of generated anti-NA mAbs; 5-12, 5-16, 6-23.1, and 6-23.2, a well-defined anti-NA antibody (1G01) was used as a positive control, and an irrelevant antibody (anti-H5) was used as a negative control. Two hours after the mAb administration, mice were intranasally infected with five times the 50% lethal dose (LD_50_) of the Ind11 H3N2 virus under anesthesia, and weight loss was monitored for 14 days postinfection. Mice that lost 25% or more of their initial body weight were humanely euthanized. The sample size for the indicated groups was: 5-12 (*n* = 4), 5-16 (*n* = 5), 6-23.1 (*n* = 3), 6.23-2 (*n* = 5), 1G01 (*n* = 5), and anti-H5 (*n* = 5).

### Cryo-EM analysis of mAb complexes: Preparation of mAb NA complex grids

Ind11 NA was incubated overnight at 4°C with 2× molar excess of mAb IgG per protomer at a final concentration of 0.6 mg/ml in TBS buffer. The 1.2/1.3 copper Quantifoil 300 mesh grid (Quantifoil Micro Tools GmbH) was used for sample vitrification. The grids were treated with Ar/O_2_ plasma (Solarus 950 plasma cleaner, Gatan) for 30 s at 15 mA immediately before sample application. A 0.5 μl of 0.7% (w/v) OBG detergent was added to 3 μl of the complex, and 3 μl was immediately loaded onto the grid. Grids were prepared using Vitrobot mark IV (Thermo Fisher Scientific).Temperature inside the chamber was maintained at 4°C, while humidity was at 100%. Blotting force was set to 1 and wait time to 5 s, while the blotting time was varied within the range of 3.5 to 5 s. Following the blotting step, the grids were plunge-frozen into liquid ethane and cooled by liquid nitrogen.

### Cryo-EM analysis of mAb complexes: Data collection and processing

Complexes were imaged at ×190,000 nominal magnification using a Falcon 4i camera on a Glacios microscope at 200 kV. Automated image collection was performed using EPU 3.5.1 (Thermo Fisher Scientific). Images were aligned, dose-weighted, and CTF-corrected in the CryoSPARC Live (*49*) software platform. Briefly, particles were picked using templates generated from the live 2D classification job and extracted at a box size of 512 pixel, Fourier cropped to 256 pixel. Clean particle stacks were selected through reference-free 2D classification, which were then used to generate a 3D reference model from ab initio job, which was then initially refined using either nonuniform refinement or homogenous refinement with C4 symmetry. Final maps were generated with C4 symmetry using local refinement in CryoSPARC 4.5.1 (*49*). The number of initial and final particles is mentioned in table S1.

### Model building and refinement

A NA homology model was used generated using Alphafold 2 (*54*) as an initial model for NA, and mAb models were generated using Abodybuilder-2 (*29*). The initial models were first rigid body fitted into the cryo-EM maps in ChimeraX (*53*), and the fitting was validated in COOT 0.9.8.7 (*30*). The models were then refined using real-space refinement in PHENIX (*55*) and COOT iteratively. The model refinement statistics are shown in fig. S5. The Kabat numbering system was used for the mAbs, and the N2 numbering convention is used for the alignment of NA.
