# Supplementary material for: Functional and epitope specific monoclonal antibody discovery directly from immune sera using cryo-EM
Source: Sci Adv. 2025 Aug 15;11(33):eadv8257. doi: 10.1126/sciadv.adv8257 (PMC12356236; doi:10.1126/sciadv.adv8257)
Supplement: Supplementary file 1 — Figs. S1 to S8 Table S1 [file sciadv.adv8257_sm.pdf]

Science Advances
AAAS

Supplementary Materials for

Functional and epitope specific monoclonal antibody discovery directly from immune sera using cryo-EM

James A. Ferguson *et al.*

Corresponding author: Andrew B. Ward, andrew@scripps.edu

Sci. Adv. **11**, eadv8257 (2025)
DOI: 10.1126/sciadv.adv8257

This PDF file includes:

Figs. S1 to S8
Table S1

pAb name	CryoEM map	MA no seq model	Chains used for HMMER search
**pAbC-1**	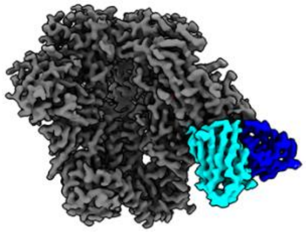	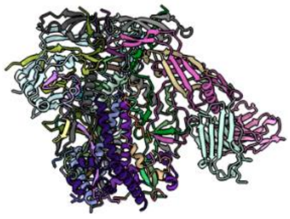	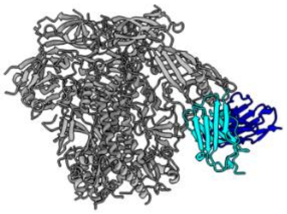
**pAbC-2**	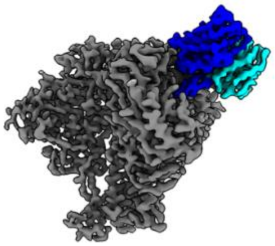	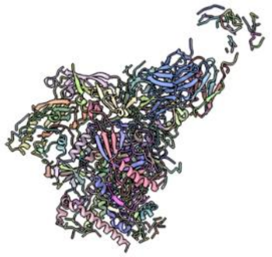	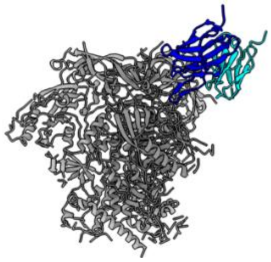

Supplemental Figure S1. Results of MA build_no_seq for pAbC-1 and pAbC-2. The cryoEM map column shows the input map used with the antigen regions shown in gray and regions corresponding to the heavy and light chain shown in dark blue and cyan, respectively. MA no seq model columns shows the output.cif generated by MA. For pAbC-2 chains corresponding to heavy and light chain were fragmented and were traced by docking in a polyA fv model. The last column shows the final chains, with corresponding .hmm files, used in the HMMER search (heavy and light are in blue and cyan respectively).

```
PABC-1_ORIGINAL_HC      QVQLQESGPGLVKPSETLSLTCAVSGGSFSGYSWGWIRQPPGKGLEWIGSIIGRTGSTAY   60
PABC-1_MA_OUTPUT_HC     SVQIQITGEDLVKPDKTLTVTCSVEGGGHSGYSYGYVRQPPGKGLEYIGMIIGRSGETDY   60
                        .**:* :* .****.:**::**:*.**..****:*::*********:** ****:*.* *

PABC-1_ORIGINAL_HC      NPSLTSRVTISRDTSNNQFSLKLTSLTAADTAVYYCARQQSNFDFWGQGVLVTVSS-      116
PABC-1_MA_OUTPUT_HC     NPKLEPRVTISRDESKNQYSLKLTDVTSGDNGVYYCGRQQAIFDYWGQGLLVTRSDV      117
                        **.*  ******* *:**:*****.:*:.*..****.***: **:****:*** *.
```

```
PABC-1_ORIGINAL_LC      DIQMTQSPSSLSASVGDTVTTTCRASQDISNDLAWYQQKPGKAPKPLLYYASNLESGVPS   60
PABC-1_MA_OUTPUT_LC     AINMTQSPTTLSGEKGETVTCTCRATQEITNDLAWFQQKPGKSPKPLIYRASNLEVGVPS   60
                         *:*****::**.. *:*** ****:*:*:*****:******:****:* ***** ****

PABC-1_ORIGINAL_LC      MFSGSGSGTDFTLTISSLQPEDFASYFCQQYNSYPRTFGQGTKVEIK         107
PABC-1_MA_OUTPUT_LC     RYSGTGSGDEFTLTISDLNPESRAIYYCMNHHERPWVFGPGTKISNR         107
                         :**:*** :******.*:**. * *:* :::. * .** ***:. :
```

```
PABC-2_ORIGINAL_HC      QVQLVQSGAEVKMPGTSVKLSCKTSGYTFTSYNINWVRQAPG-QALEWMGWINPNNGTTD   59
PABC-2_MA_OUTPUT_HC     NRQLTQAGSAVKKPGESVKLSCKAAGRNFSAYNINWVRQADGKQALEWMGYLNPENGQEE   60
                        : **.*:*: ** ** *******::* .*::********* * *******::**:**  :

PABC-2_ORIGINAL_HC      YAQKFQGRVTMTRDTSTTTAYMQLNSLRSEDTAVYYCARARGGYEDDDGYHYTGYGLDSW   119
PABC-2_MA_OUTPUT_HC     YSEEFEGRVTFSRDTETNEVYLQLKNLKVENTSIYYCARARAGYEDEEGFHYTGYGMDTF   120
                        *:::*:****::***.*. .*:**:.*: *:*::*******.****::*:******:*::

PABC-2_ORIGINAL_HC      GQGVVVTVSS-    129
PABC-2_MA_OUTPUT_HC     GQAVVIEVSPS    131
                        **.**: **
```

```
PABC-2_ORIGINAL_LC      --DIQMTQSPSSLSASIGDRVTVTCRASQGINMQLCWYQLKPGKAPTLLIYGTSGLQTGV   58
PABC-2_MA_OUTPUT_LC     GEKIKMTQSPSXXSSSLGDRVTVTCRAAEGNENELSWYKQLPGKPPTLLIYGADGINSGV   60
                          .*:******  *:*:**********::* : :*.**:  *** *******:.*:::**

PABC-2_ORIGINAL_LC      SSRFSGSGSGTNFTLTISSLQ-PEDVATYYCQQDYTTPFTFGPGTKLDIK--   107
PABC-2_MA_OUTPUT_LC     SPRFSGSGGDNDFSLTLSSLNNPNDIGVFYQMQCHSPVFTFGPGVEVPEGLS   112
                        * ******...:*:**:***: *:*:..:*  * ::  ******.::
```

Supplemental Figure S2. Sequence identity comparisons of MA build_no_seq model output vs mAbs originally identified from manual structure to sequence(SFS) paper.

```
PABC-1_ORIGINAL_HC      QVQLQESGPGLVKPSETLSLTCAVSGGSFSGYSWGWIRQPPGKGLEWIGSIIGRTGSTAY    60
PABC-1_TOPHIT_HC        QVQLQESGPGLVKPSETLSLTCAVSGGSFSGYSWGWIRQPPGKGLEWIGYIIGRTGSTDY    60
                        ************************************************* ******** *

PABC-1_ORIGINAL_HC      NPSLTSRVTISRDTSNNQFSLKLTSLTAADTAVYYCARQQSNFDFWGQGVLVTVSS        116
PABC-1_TOPHIT_HC        NPSLTSRVTISRDTSNNQFSLKLTSLTAADTAVYYCARQQSNFDFWGQGVLVTVSS        116
                        ********************************************************
```

```
PABC-1_ORIGINAL_LC      DIQMTQSPSSLSASVGDTVTTTCRASQDISNDLAWYQQKPGKAPKPLLYYASNLESGVPS    60
PABC-1_TOPHIT_LC        DIQMTQSPSSLSASVGDTVTATCRASQDITNDLAWYQQKPGKAPKPLIYYASNLESGVPS    60
                        ********************:********:*****************:************

PABC-1_ORIGINAL_LC      MFSGSGSGTDFTLTISSLQPEDFASYFCQQYNSYPRTFGQGTKVEIK 107
PABC-1_TOPHIT_LC        RFSGSGSGTDFTLTISSLQPEDFAIYFCQQFYTYPRTFGQGTKVEIK 107
                         *********************** *****: :**************
```

```
PABC-2_ORIGINAL_HC      QVQLVQSGAEVKMPGTSVKLSCKTSGYTFTSYNINWVRQAPGQALEWMGWINPNNGTTDY    60
PABC-2_TOPHIT_HC        QVQLVQSGAEVKMPGTSVKLSCKTSGYTFTSYNINWVRQAPGQALEWMGWINPKNGKTDY    60
                        *****************************************************:**.***

PABC-2_ORIGINAL_HC      AQKFQGRVTMTRDTSTTTAYMQLNSLRSEDTAVYYCARARGGYEDDDGYHYTGYGLDSWG    120
PABC-2_TOPHIT_HC        AKKFQGRVTMTRDTSTTTAYMQLNSLRSEDTAVYYCARARGGYEDDDGYHYTGYGLDSWG    120
                        *:**********************************************************

PABC-2_ORIGINAL_HC      QGVVVTVSS       129
PABC-2_TOPHIT_HC        QGVVVTVSS       129
                        *********
```

```
PABC-2_ORIGINAL_LC      DIQMTQSPSSLSASIGDRVTVTCRASQGINMQLCWYQLKPGKAPTLLIYGTSGLQTGVSS    60
PABC-2_TOPHIT_LC        DIQMTQSPSSLSASIGDRVTVTCRASQGIKKELSWFQQRPGRAPTLLIYGASSLQTGVST    60
                        *****************************: :*.*:* :**:********:*.******:

PABC-2_ORIGINAL_LC      RFSGSGSGTNFTLTISSLQPEDVATYYCQQDYTTPFTFGPGTKLDIK 107
PABC-2_TOPHIT_LC        RFSGSGSGTDFTLTISSLQPEDVATYYCQQDFSPPFTFGVGTKVEIK 107
                        *********:*********************:: ***** ***::**
```

Supplemental Figure S3. Sequence identity comparisons of ModelAngelo top hits from HMMER vs mAbs originally identified from manual SFS paper.

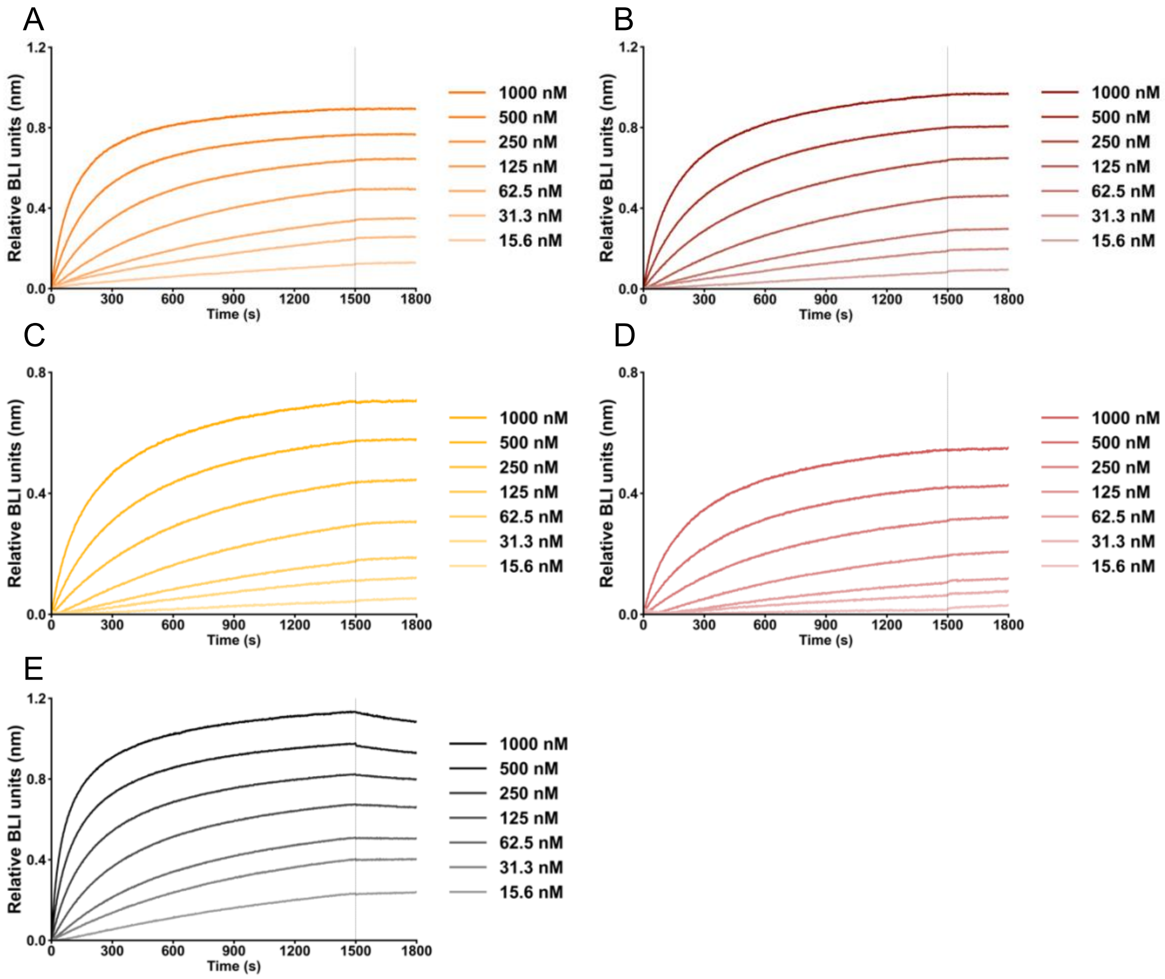

Supplemental Figure S4. Complete dilution series of BLI binding curves for determination of kinetic binding parameters of interactions between BG505 SOSIP (A) pAbC-1_ModelAngelo (B) pAbC-1_Manually derived (C) pAbC-2_ModelAngelo (D) pAbC-2_Manually derived (E) PGT-145.

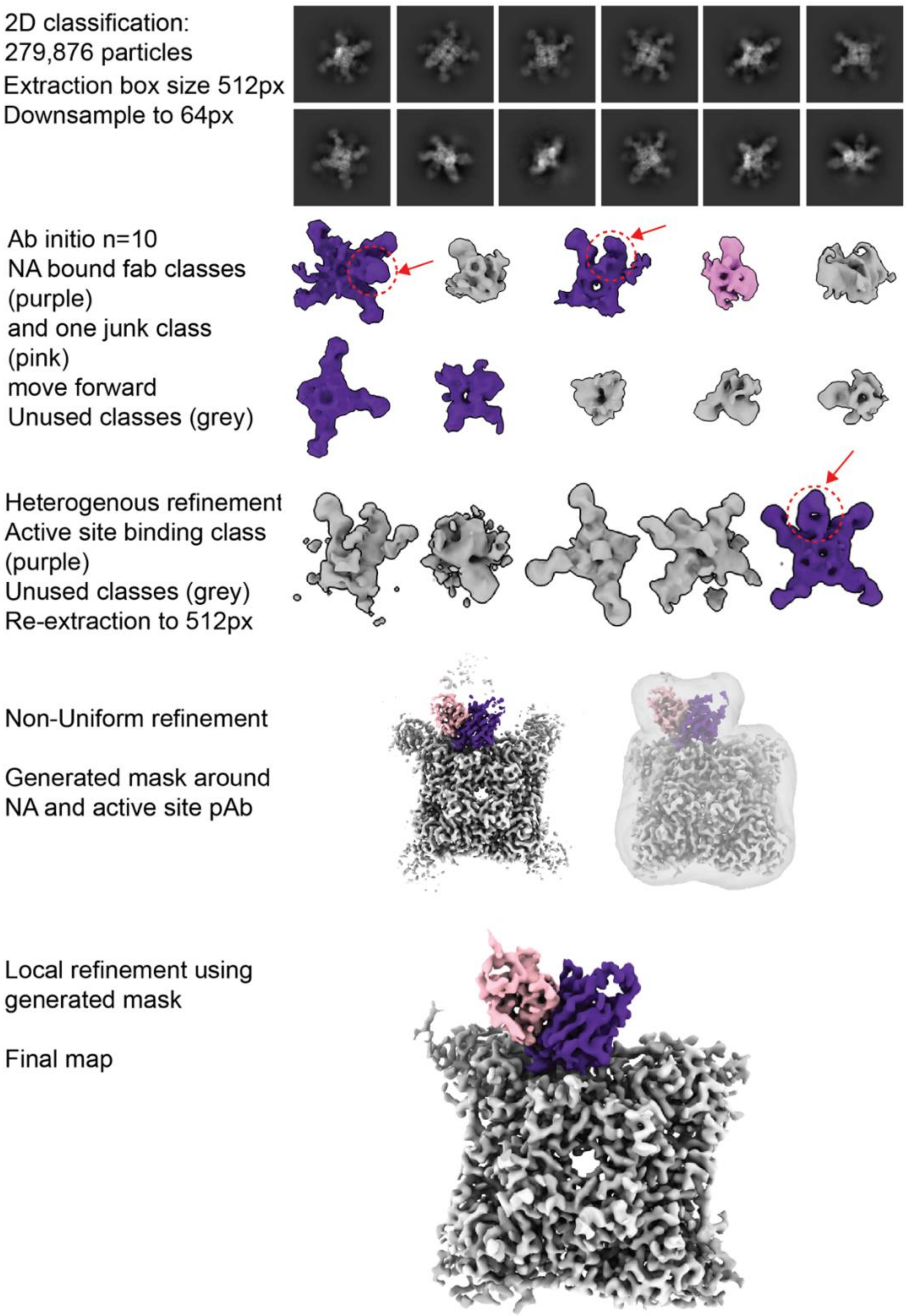

Supplemental Figure S5. cryoEMPEM processing workflow in cryosparc v4.3.0

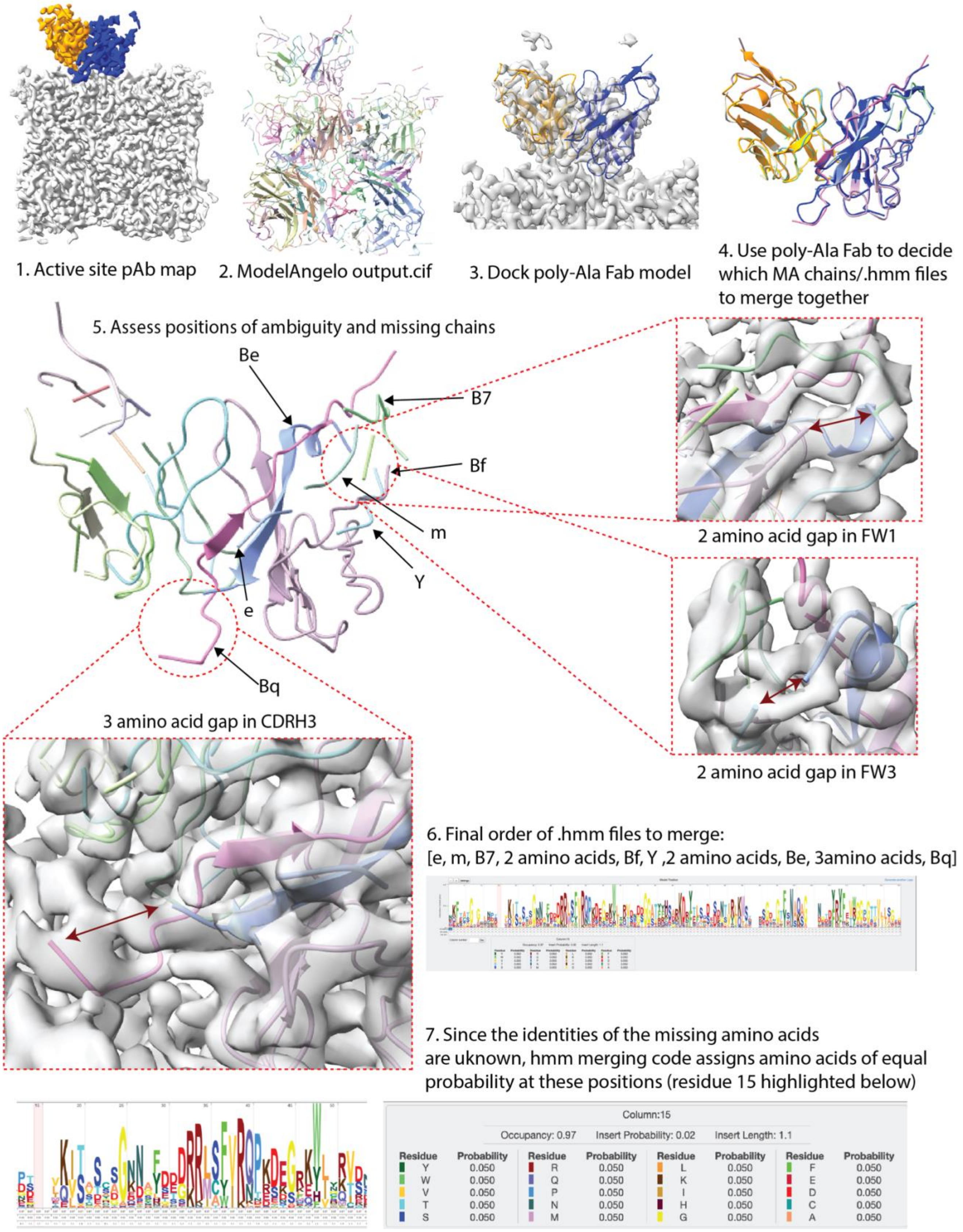

Supplemental Figure S6. Representative ModelAngelo Sequence From Structure workflow.

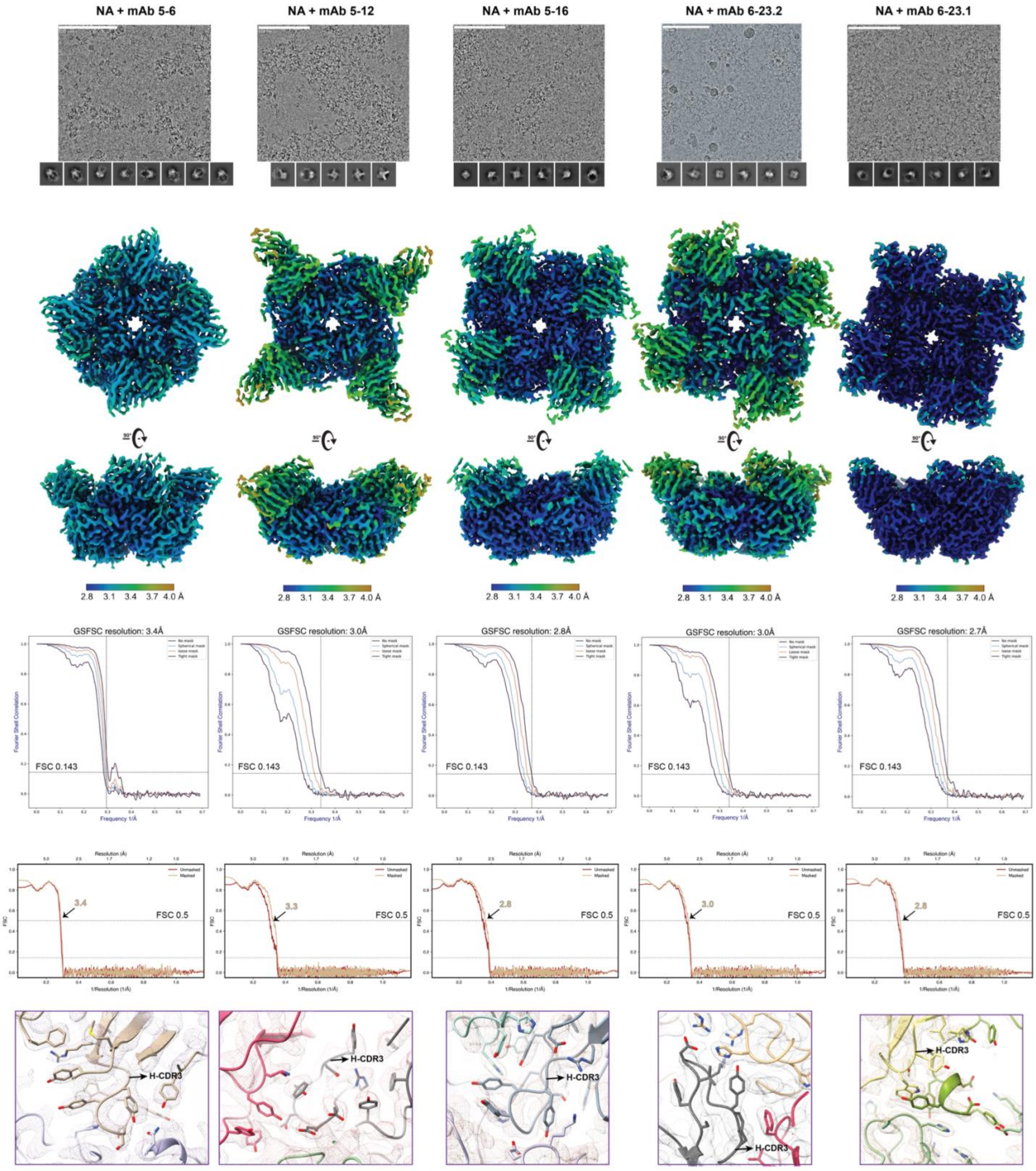

Supplemental Figure S7. Representative micrographs, 2D class averages, local resolution maps, FSC curves, and CDRH3s with electron potential maps for each NA-inhibiting mAb.

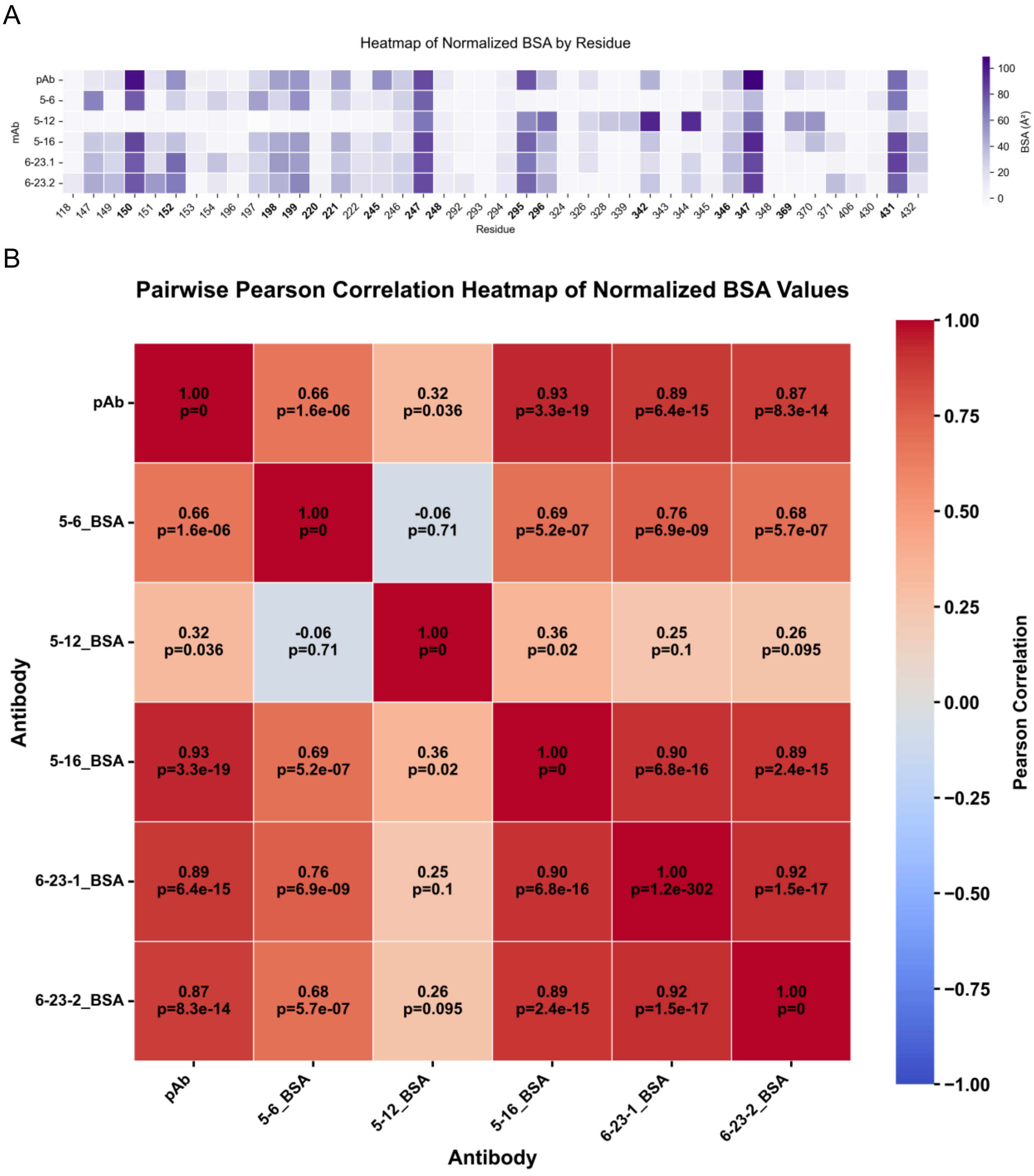

Supplemental Figure S8. NA mAb to pAb structural comparisons. A. Normalized heat map of antibody contributions to BSA of pAb vs the five isolated mAbs. Residues in bold correspond to pAb interaction residues. B. Pearson correlation coefficient of each mAb BSA per residue.

	NA in complex with polyclonal antibody (EMDB-48118)	NA in complex with 5-6 (EMDB-48165) (PDB-9MD2)	NA in complex with 5-12 (EMDB-48166) (PDB-9MD3)	NA in complex with 5-16 (EMDB-48167) (PDB-9MD4)	NA in complex with 6-23.2 (EMDB-48168) (PDB-9MD5)	NA in complex with 6-23.1 (EMDB-48169) (PDB-9MD6)
**Data collection and processing**						
Magnification	190000	190000	190000	190000	190000	190000
Voltage (kV)	200	200	200	200	200	200
Electron exposure (e–/Å^2)	43.4	45	45	45.1	45.12	40.9
Defocus range (μm)	-1.8 to -0.8	-1.8 to -0.8	-1.8 to -0.8	-1.8 to -0.8	-1.8 to -0.8	-1.8 to -0.8
Pixel size (Å)	0.725	0.725	0.725	0.725	0.725	0.718
Symmetry imposed	C1	C4	C4	C4	C4	C4
Initial particle images (no.)	436610	593280	740197	474585	254209	561753
Final particle images (no.)	72780	79388	67241	117508	77151	91670
Map resolution (Å) FSC threshold (0.143)	3.3	3.4	2.9	2.7	2.9	2.7
**Refinement**						
Model resolution (Å) FSC threshold (0.5)	-	3.4	3.3	2.8	3.0	2.8
Map sharpening *B* factor (Å^2)	-	-115.8	-71.6	-91.3	-74.5	-76.3
Map Correlation Coefficient (Mask)	-	0.85	0.87	0.89	0.87	0.89
Model composition						
Non-hydrogen atoms	-	19540	20020	19688	19884	19532
Protein residues	-	2468	2468	2472	2472	2468
Ligands	-	CA:8	CA:4	CA:4	CA:4	CA:4
	-	NAG:24	NAG:40	NAG:24	NAG:32	NAG:20
	-	BMA:4 MAN:8	BMA:8 MAN:20	BMA:4 MAN:8	BMA:4 MAN:8	BMA:4 MAN:8
B factors (Å^2)						
Protein	-	18.66	19.50	28.41	22.51	18.57
Ligand	-	13.91	23.04	28.04	23.02	32.83
R.m.s. deviations						
Bond lengths (Å)	-	0.004	0.002	0.003	0.006	0.002
Bond angles (°)	-	0.968	0.578	0.618	0.756	0.560
Validation						
MolProbity score	-	1.45	1.45	1.43	1.74	1.3
Clashscore	-	8.02	7.89	7.86	9.12	5.6
Poor rotamers (%)	-	0	0	0	0	0
Cβ outliers	-	0	0	0	0	0
Ramachandran plot						
Favored (%)	-	97.95	97.91	98.20	96.2	98.69
Allowed (%)	-	1.88	1.92	1.47	3.64	1.15
Disallowed (%)	-	0.16	0.16	0.33	0.16	0.16

Supplemental Table S1. Table of statistics for pAb map and mAbs
